# The cancer-obesity connection: what do we know and what can we do?

**DOI:** 10.1186/1741-7007-12-9

**Published:** 2014-02-04

**Authors:** Elio Riboli

**Affiliations:** 1 The School of Public Health, Imperial College London, Norfolk Place, London W2 1PG, UK

##  

Elio Riboli holds a medical degree from the State University of Milan and a Master’s degree in public health from Milan, and took a Master’s degree in Science in Epidemiology at Harvard University. From 1983 to 2005 he was the Head of the Nutrition, Hormone and Cancer Unit at the International Agency for Research on Cancer, and is now Director of the School of Public Health at Imperial College London, where his research has focused on the role of nutrition and metabolism in the etiology of cancer. He has led since 1990 the European Prospective Investigation into Cancer (EPIC) that has enrolled half a million study participants from 10 European countries.

**Figure:**
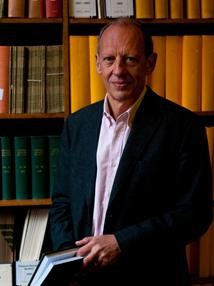
Elio Riboli

## Is the evidence for a link between cancer and obesity now compelling?

I would say yes. I would say that there is sufficient evidence to support a causal link between obesity and a number of cancers.

## So it’s not all cancers?

It’s not all cancers. For some cancers, the association has been observed quite consistently in different studies and in different populations. For other cancers, the association has been - the results have been - less consistent. And possibly there are quite a number of cancers that are simply not associated with obesity.

## So which are the cancers that you would say are, on the evidence, associated with obesity - and this is epidemiological evidence that we’re talking about, isn’t it?

Yes. It’s epidemiological evidence often supported by mechanistic studies - other studies using biomarkers in humans or studies using experimental models in rodents. I would say my short-list is based on a number of consensus papers that have been published over the past year or two.

Number one, I would say, breast cancer after menopause - where after menopause is extremely important because there is no increased risk of breast cancer before menopause associated with obesity. If anything, overweight women have a slightly reduced risk of developing breast cancer during their reproductive life, or until when they enter into menopause.

## That’s very mystifying, is it not?

It is really, really a nightmare. Because the same results were found in most of the North American cohorts and by us in Europe. So observationally, if we just take the weight of women who are in their 20s or 30s and we follow them up and compare the incidence of breast cancer by looking at body mass index (BMI), it is quite clear that there is a slight reduction of breast cancer incidence among overweight and obese women, that is actually linearly associated in more obese women. We are talking about not a huge difference; we’re talking about a 10 to 15% reduced incidence in overweight-obese women in the age range 30 to 45/50.

## And after menopause, how big is the difference?

Well, it reverses. I’d say a relative risk of 1.2, 1.4, anything between 20 and 30% increased risk.

## So not huge, again?

It’s not huge, but for a very highly prevalent cancer like breast cancer - where 10 women out of 100 will develop the cancer in their life in most European countries - it says that probably 2 out of 10 of those cancers might be prevented if women would not be overweight - if they would stay on a BMI below 26. The other cancers that have been consistently found at higher risk in overweight and obese subjects are cancer of the colon and rectum, as well as cancer of the endometrium - which is probably the cancer which has the strongest association of all with obesity - I would say it was even known 20 years ago that obesity increases the risk of endometrial cancer, it doesn’t matter at which age - at all possible ages. And another is kidney cancer, where there is an increased risk in people who are overweight and - to complicate things - in people who have hypertension. This has been found in both European and North American studies.

Finally, but it’s not trivial, there is a very clear association of obesity with the adenocarcinoma of the oesophagus. I’m sure you are familiar with the fact that oesophagus cancer comes in two main types. One is called the squamous cell carcinoma, which is strongly related to alcohol and tobacco consumption. The other is the one that - when I started 20 or 30 years ago - was extremely rare in North America and Europe: adenocarcinoma. Now, this adenocarcinoma of the oesophagus, which represented maybe a maximum of 5% or 10% of the cases 30 years ago, now represents over 50% of 60% of the cases of oesophageal cancer in North America and Europe. This is the one that, together with cancer of the upper part of the stomach - called the cardia - it’s the upper part of the stomach, close to the oesophageal connection - these cancers, these adenocarcinomas, are associated with obesity. There are very consistent results on this.

So to summarize, it’s breast post-menopausal, colorectum, endometrium, kidney, and then adenocarcinoma of the oesophagus and the cardia. There are limited data, which give more conflicting results, regarding aggressive advanced prostate cancer, for which some studies found an association, but others don’t find an increased risk. And a little bit more, let’s say, discordant results for cancer of the pancreas. If, overall, one looks at cancer mortality - as was done, for example, in the follow-up of the huge American Cancer Society cohort - what you see is that overall obesity is associated with overall increased mortality for cancer. But it’s mainly due to these cancers.

## Why should endometrial cancer be particularly strongly associated with obesity?

Essentially, endometrial cancer is very strongly associated with estrogen levels. So we have published from our EPIC study, and others have published from other studies, data showing that there is a very strong association: the higher the blood estrogen levels, the higher the risk of endometrial cancer. The association is very, very strong. Within what is called the ‘normal physiological range’ of estrogen levels in blood, women with the highest levels have increased risk of endometrial cancer. It’s something like three- to four-fold.

Now, if then you look physiologically, what is absolutely crystal clear is that BMI is linearly associated with estrogen levels in the blood. So the more adipose tissue there is, the more estrogens. The mechanism is very well known, because adipose tissue is very rich in an enzyme called aromatase. The aromatase converts androgens into estrogens. Basically, the adipose tissue is a very metabolically active tissue - contrary to what one may think - and has this important property of producing estrogens.

## So that fully explains that relationship?

It’s the main explanation of the relationship. The other one is insulin resistance, which is the one that we have been working on for more than a decade. Women who are insulin resistant tend, obviously, to have higher levels of insulin, because they need more insulin to use their glucose. When blood levels of insulin go up, there is a negative feedback on the liver production of a globulin that has a very central role in regulating sex hormones. It’s called sex hormone binding globulin - SHBG.

The sex hormone binding globulin binds, in an active form, estrogens and androgens and is down-regulated by insulin. When people overeat and develop insulin resistance, are overweight, and have low physical activity - they tend to have high blood levels of insulin. Insulin will then inhibit the liver production of sex hormone binding globulin, and that will result in an increased proportion of free estrogens and free androgens.

So there are two effects. One is that in the obese, the adipose tissue produces estrogens, converts androgens to estrogens. The second one is that insulin, down-regulating sex hormone binding globulin, makes estrogen more bioavailable.

## So it’s actually the sex hormones that are to blame in both cases?

Yes… and this is supported by metabolic and experimental models.

## That brings us to the question: if there is a clear link between obesity and cancers of various kinds, can they be prevented by diet and are there any particular dietary items or is it just a question of excessive intake of food?

That is actually the most difficult question to answer. Certainly the component of cancer risk that is due to obesity is the end result of what, in nutrition, we call ‘energy balance’, which is the balance between our energy expenditure and our energy intake. So the effect is due to obesity. But the research conducted over the past two decades indicates that there is also - I would call it - almost a separate parallel effect of the type of food, with results that are quite well known in the scientific but also in the lay press. Generally, consumption of red meat and processed meat is associated with a modest but consistently observed increased risk of developing cancer of the colon and rectum, specifically. Consumption of fruit and vegetables has a much weaker effect than we thought in the past, but a diet rich in fruit and vegetable and with relatively modest intake of red meat is generally associated with a decreased risk of cancer of different types.

Now, if anything has changed over the past 10 years, it’s that 10 years ago, if you had asked the same question of me and many other colleagues, we would have said: ‘Well, the main association of cancer and nutrition is via the type of food you eat’. Over the past decade, the evidence of the association of the effect of obesity has come up very, very strongly. And I should say - obesity AND lack of physical activity, almost equally. This has been a switch in our understanding of the relationship between nutrition and cancer - the emerging role of obesity and sedentary life.

## That raises the crucial practical question of whether there is any way that this can realistically be tackled, in the way that smoking was tackled some decades ago?

This is the nightmare question because clearly there has been growing knowledge and understanding that - whether it’s for cancer or diabetes or cardiovascular diseases - obesity is not a good thing for health. Despite widespread knowledge, there are impressive data on the epidemic of obesity around the world [1,2] that show the prevalence of obesity in many parts of the world continues to increase. So whatever we are saying and whatever we are trying to do is not working.

What we have is the knowledge that if you set up a very intense weight loss program, people generally tend to lose weight. The big problem is that when people are out of that program - after 6 months or 12 months, they’ve lost their few kilograms - weight maintenance becomes the nightmare. Because people lose weight and then progressively tend to come back to the way that they were before. It’s the so-called yo-yo function.

It’s a big challenge we have now from a public health point of view, clearly. If you focus on just one individual, you may win the battle. But when you think there are over two billion people overweight or obese around the planet, then it’s a serious matter.

## Do you think there’s any hope?

Well, I don’t know. Having been in cancer epidemiology for almost three decades now, I would say, I see where we are with obesity now, with due, let’s say, prudence, is where we were with smoking in the 1970s. Everybody knew that smoking was bad. Everybody knew it was bad, but people were smoking everywhere! In the streets, in the restaurants, in the pubs, in the bars, in the theatres.

People were smoking in hospitals. Hospitals! When my first son was born, I spoke with the obstetrician and he had a pack of Marlboro in his hand, and I said, ‘Don’t you recommend pregnant women to stop smoking?’ And he said: ‘But why should *I*?’

I’ll try to put a pinch of optimism in our conversation. In the 1970s, we knew that smoking was really bad but it looked like the tobacco industry was unbeatable. Now we have come a long way. We’ve clearly shown that smoking habits are really changing now, have dropped enormously in Europe and North America and Australia. So we can win the battle. Maybe it’s a little bit like that now with obesity. The huge social class gradient in obesity suggests that with education, with better understanding of the risk factors and so on, maybe it’s going to work. Because also smoking started decreasing in the higher social class and then spread to the entire society - I mean more and more people started giving up smoking.

## And also there was pressure back on the tobacco companies. Would we not also have to tackle the people who make popular high-sugar foods and drinks?

Yes, I think certainly there are issues here as well. Pressure for some sectors in the food industry that we know extremely well promote foods which are very rich in what we call the void calories - where there is an excessive amount of sugar and fat, while the same food could be produced in a more healthy way. Or rather than sweet drinks, people may just drink something else. Water, for example!

## Wouldn’t it be nice to see the same thing happen to high-carbohydrate, high-sugar foods that happened to smoking?

I’ll be very happy to see that change in trend.
